# Evaluation of the correlation between quantitative measurement of the foveal avascular zone and retinal vessel density and outer retinal disruptions in diabetic patients

**DOI:** 10.3906/sag-1901-22

**Published:** 2019-08-08

**Authors:** Nagihan UĞURLU*, Ayşe Güzin TAŞLIPINAR UZEL, Ahmet ŞENGÜN, Fatma YÜLEK, Demet ÖZDAŞ, Ali Abbas TAM, Reyhan ERSOY, Bekir ÇAKIR

**Affiliations:** 1 Department of Ophthalmology, Faculty of Medicine, Ankara Yıldırım Beyazıt University, Ankara Turkey; 2 Department of Ophthalmology, Sandıklı State Hospital, Afyonkarahisar Turkey; 3 Department of Ophthalmology, Faculty of Medicine, Ufuk University, Ankara Turkey; 4 Department of Endocrinology and Metabolism, Faculty of Medicine, Ankara Yıldırım Beyazıt University, Ankara Turkey

**Keywords:** Outer retinal disruption, external limiting membrane, ellipsoid zone, interdigitation zone, foveal avascular zone, superficial capillary plexus, deep capillary plexus, optical coherence tomography angiography

## Abstract

**Background/aim:**

The aim of the current study was to evaluate the correlation between the integrity of the outer retinal layers on optical coherence tomography (OCT) and objective parameters of retinal microvascular perfusion on optical coherence tomography angiography (OCTA).

**Materials and methods:**

A total of 105 eyes of 54 diabetic patients were included in the study. Integrity of the outer retinal layers including the external limiting membrane (ELM), ellipsoid zone (EZ), and interdigitation zone (IZ) was assessed by spectral-domain optical coherence tomography. The foveal avascular zone (FAZ) area and vessel density (VD) measurements in the superficial capillary plexus (SCP) and deep capillary plexus (DCP) in all the Early Treatment Diabetic Retinopathy Study (ETDRS) sectors were evaluated by OCTA. Associations between the quantitative measurement of the FAZ and retinal VD measurements and outer retinal disruptions were evaluated.

**Results:**

The FAZ area was correlated with outer retinal layer disruption both in the superficial plexus (r = 0.244, 0.228, 0.212, P = 0.013, 0.02, 0.031 for the ELM, EZ, and IZ, respectively) and the deep capillary plexus (r = 0.298, 0.234, 0.197, P = 0.002, 0.019, 0.048 for the ELM, EZ, and IZ, respectively). A significant relationship was also found between the VD measurements in the SCP and DCP in ETDRS sectors and the outer retinal layers disruption.

**Conclusion:**

The results of the current study show a significant relationship between the quantitative OCTA parameters and the integrity of the outer retinal layers. This finding reveals a correlation between retinal capillary nonperfusion and outer retinal disruption in eyes with diabetic retinopathy.

## 1. Introduction

The outer retina includes foveal photoreceptors and their connections to the Müller cells and retinal pigment epithelium cells [1]. The external limiting membrane (ELM), ellipsoid zone (EZ), and interdigitation zone (IZ) are the first three hyperreflective bands of the outer retina in optical coherence tomography (OCT) sections and are frequently used in the evaluation of foveal photoreceptor health and integrity [2]. Disruptions in the ELM, EZ, and IZ are known to be associated with various degenerative or inﬂammatory chorioretinal diseases [3,4].

The foveal photoreceptor cells have a high demand for oxygen and nutrients [5]. This high oxygen and nutrient requirement is normally met by a complex vascular supply [6]. When the vascular supply is impaired and fails to meet the needs of photoreceptors, structural and functional destruction of the photoreceptor layers occurs [7,8].

The avascular outer retina is supplied mainly by diffusion from the choroidal circulation and to a lesser extent by the retinal circulation [9]. The role of the retinal circulation in photoreceptor oxygenation may increase in hypoxic conditions [10]. This has led to the notion that retinal perfusion destruction may damage the photoreceptor morphology and integrity in chronic hypoxic conditions such as diabetes. There are studies showing photoreceptor damage in cases with deep capillary plexus (DCP) defects [6]. Other studies have also shown a relationship between outer retinal damage and DCP injury [11,12]. However, no study has evaluated the relationship between quantiﬁed measurement of retinal microvasculature at the level of both the superficial capillary plexus (SCP) and DCP and FAZ measurements and outer retinal disruption in diabetic retinopathy patients.

Optical coherence tomography angiography (OCTA) is a new technology that allows the imaging of the retinal microvascular structure at the cellular level in a noninvasive manner and with high reliability and reproducibility [13]. In addition, the innovations in the software providing automated quantification algorithms also allow objective grading of retinal microvascular perfusion in the Early Treatment Diabetic Retinopathy Study (ETDRS) sectors at the levels of the SCP and DCP [14].

The aim of the current study was to investigate the relationship between the integrity of the ELM, EZ, and IZ and OCTA results, retinal vessel density (VD), and foveal avascular zone (FAZ) dimensions at the SCP and DCP in diabetic retinopathy (DR).

## 2. Materials and methods

The study protocol was approved by Ankara Yıldırım Beyazıt University and complied with the tenets of the Declaration of Helsinki. The study included 105 eyes of 54 diabetic patients followed at the Diabetic Eye Disease Center. The diagnosis of DR was made according to the ETDRS criteria by taking into consideration the OCT during the examination and the FFA findings in the required cases [15]. According to the classification, two groups were included into the study: no DR (eyes with no signs of DR) and DR (eyes at different stages of DR without macular edema). The following subjects were excluded: patients with diabetic macular edema (DME), spherical equivalent of more than ±1 diopter, history of intraocular surgery, or systemic disease other than DM, in addition to those younger than 18 years of age or those who were pregnant. 

**Figure 1:**
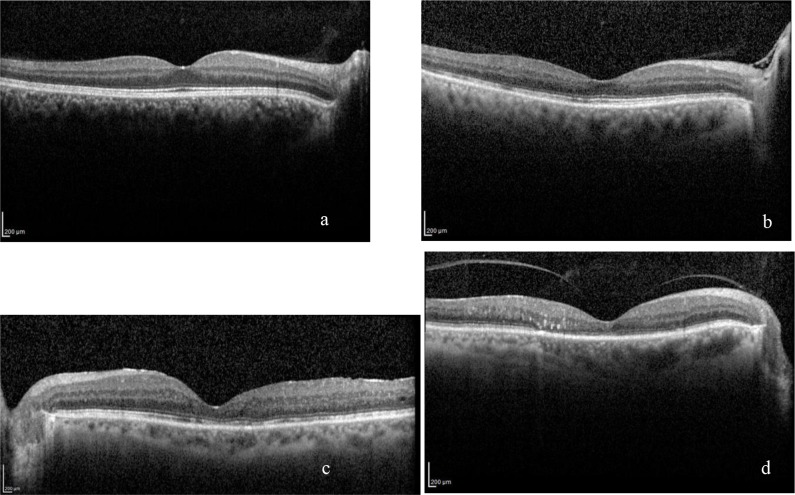
Examples demonstrating the grading of ELM, EZ, and IZ band disruptions on SC-OCT images. a) ELM Grade 0, EZ Grade 0, IZ Grade 0; b) ELM Grade 0, EZ Grade 1, IZ Grade 2; c) ELM Grade 0, EZ Grade 2, IZ Grade 2; d) ELM Grade 2, EZ Grade 2, IZ Grade 2.

After fundus evaluation by two ophthalmologists who were not aware of the other’s decision, OCT images (Heidelberg Spectralis, Heidelberg, Germany) with cross-sectional spectral-domain optical coherence tomography (SD-OCT) scans (30° × 15° with 19 horizontal sections, ART 9) were obtained. Integrity of the ELM, EZ, and IZ was defined as the continuous back-reflection line corresponding to the respective lines. Disruptions of the ELM, EZ, and IZ were assessed in each image for 500 µm in either direction of the fovea along the horizontal and vertical scans by the same two ophthalmologists. The line disruption was graded from 0 to 2. Grade 0 bands had no disruption, grade 1 bands had focal disruptions of ≤200 µm, and grade 2 bands had focal disruptions of ˃200 µm (Figures 1a–1d).

An RTVue XR Avanti device (Optovue Inc, Fremont, CA, USA) using the split-spectrum amplitude-decorrelation angiography algorithm was used for the OCTA images. An A-scan rate of 70,000 scans per second with a light source centered at 840 nm and a bandwidth of 50 nm was used. To produce three-dimensional angiograms, each B-scan contained 316 A-scans in the 3 × 3 mm central area of the macula. Motion correction was performed using the recording of two orthogonally captured imaging volumes in 3 s, due to microsaccades and fixation changes.

The image was set at 15 µm beneath the inner plexiform layer to obtain images of the superficial vascular layers (superficial plexus). To obtain images of the deep vascular layers (deep plexus), the image was segmented with an inner boundary at 15 µm and the outer boundary at 70 µm beneath the inner plexiform layer. Vessel density and FAZ measurements were evaluated as previously described [14]. Flow areas, nonflow areas, and vessel densities of the fovea, parafovea, superior hemisphere, inferior hemisphere, the sectors (superior, temporal, inferior, and nasal), and the grid based on the ETDRS chart where the fovea was centered were analyzed for each plexus. 

Data were analyzed using SPSS 21 (IBM Corp., Armonk, NY, USA). P < 0.05 was accepted as statistically significant. The Shapiro–Wilk test was used to verify the normality of the distribution. The Mann–Whitney U test and Spearman correlation test were used for analyzing nonparametric data. 

## 3. Results

In this study, 70 eyes of 36 patients in group 1 (diabetic patients without DR) and 35 eyes of 18 patients in group 2 (diabetic patients with DR) were analyzed. Mean age and sex ratio (female/male) of group 1 were 55.5 ± 13.7 years and 36/34, while these were 57.8 ± 11.1 years and 12/23 in group 2 (P = 0.838 and 0.072, respectively). The duration of DM and mean HbA1c level were 11.43 ± 5 years and 7.49 ± 1.46 in group 1 and 12.71 ± 5 years and 7.87 ± 1.17 in group 2 (P = 0.159 and 0.108, respectively).

Mean visual acuity was 0.14 ± 0.15 logMAR in group 1 and 0.29 ± 0.27 logMAR in group 2 (P ˂ 0.01). Correlations of visual acuity and parameters of OCTA are shown in Table 1.

**Table 1:** Correlation of visual acuity and OCTA parameters.

		r	P
Superficial capillary plexus	FAZ	0.395	P < 0.01
	Global VD	–0.615	P < 0.01
	Temporal VD	–0.579	P < 0.01
	Superior VD	–0.547	P < 0.01
	Nasal VD	–0.614	P < 0.01
	Inferior VD	–0.571	P < 0.01
Deep capillary plexus	FAZ	0.416	P < 0.01
	Global VD	–0.565	P < 0.01
	Temporal VD	–0.385	P < 0.01
	Superior VD	–0.508	P < 0.01
	Nasal VD	–0.509	P < 0.01
	Inferior VD	–0.500	P < 0.01

VD: Vessel density. FAZ: Foveal avascular zone. P < 0.05 is significant.

The FAZ of the SCP was 0.36 ± 0.58 mm2 in group 1 and 0.46 ± 0.28 mm2 in group 2 (P = 0.006). The same parameter for the DCP was 0.40 ± 0.17 mm2 in group 1 and 0.48 ± 0.20 mm2 in group 2 (P = 0.037). Table 2 shows the comparison of vessel densities of the SCP and DCP in the two groups. Table 3 shows the correlation of the FAZ and vessel densities with disruption of the ELM, EZ, and IZ. The FAZ area was correlated with outer retinal layer disruption both in the superficial plexus (r = 0.244, 0.228, 0.212, P = 0.013, 0.02, 0.031 for the ELM, EZ, and IZ, respectively) and deep capillary plexus (r = 0.298, 0.234, 0.197, P = 0.002, 0.019, 0.048 for the ELM, EZ, and IZ, respectively). There were negative correlations between global VD and disruption of the ELM, EZ, and IZ both in the superficial plexus (r = –0.273, –0.352, –0.195, P = 0.006, ˂0.01, 0.054 for the ELM, EZ, and IZ, respectively) and the deep capillary plexus (r = –0.310, –0.352, –0.355, P = 0.002, ˂0.01, ˂0.01 for the ELM, EZ, and IZ, respectively). Similar correlations were also observed between all VD measurements in the ETDRS sectors and outer retinal layer disruption (Table 3). FAZ values were correlated weakly with disruption of the ELM, EZ, and IZ. 

**Table 2:** Comparison of vessel densities in the groups.

	%	Group 1	Group 2	P
Superficial capillary plexus	Global	52.67 ± 3.46	46.76 ± 5.90	P ˂ 0.01
	Temporal	53.92 ± 3.40	48.05 ± 5.71	P ˂ 0.01
	Superior	55.42 ± 4.26	48.82 ± 7.02	P ˂ 0.01
	Nasal	54.12 ± 4.36	47.77 ± 6.98	P ˂ 0.01
	Inferior	55.73 ± 4.31	49.48 ± 7.97	P ˂ 0.01
Deep capillary plexus	Global	58.69 ± 2.95	52.97 ± 6.36	P ˂ 0.01
	Temporal	60.20 ± 4.40	55.21 ± 5.79	P ˂ 0.01
	Superior	62.61 ± 2.79	56.75 ± 7.18	P ˂ 0.01
	Nasal	60.58 ± 3.42	54.08 ± 7.13	P ˂ 0.01
	Inferior	62.61 ± 3.55	55.61 ± 9.25	P ˂ 0.01

P ˂ 0.05 is significant.

**Table 3:** Correlation of disruption of the ELM, EZ, and IZ and OCTA parameters.

		ELM		EZ		IZ	
		r	P	r	P	r	P
SCP	FAZ	0.244	0.013	0.228	0.02	0.212	0.031	Global VD	–0.273	0.006	–0.352	˂0.01	–0.195	0.054	Temporal VD	–0.289	0.004	–0.36	˂0.01	–0.233	0.021	Superior VD	–0.192	0.057	–0.358	˂0.01	–0.149	0.141	Nasal VD	–0.212	0.035	–0.328	0.001	–0.139	0.171	Inferior VD	–0.326	0.001	–0.347	˂0.01	–0.216	0.032
	DCP	FAZ	0.298	0.002	0.234	0.019	0.197	0.048	Global VD	–0.310	0.002	–0.352	˂0.01	–0.355	˂0.01	Temporal VD	–0.319	0.001	–0.353	˂0.01	–0.331	0.001	Superior VD	–0.214	0.032	–0.357	˂0.01	–0.322	0.001	Nasal VD	–0.218	0.029	–0.352	˂0.01	–0.267	0.007	Inferior VD	–0.335	0.001	–0.357	˂0.01	–0.313	0.002

SCP: Superficial capillary plexus, DCP: deep capillary plexus, VD: vessel density, FAZ: foveal avascular zone. P ˂ 0.05 is significant.

## 4. Discussion

The current study demonstrated a significant correlation between the integrity of the ELM, EZ, and IZ and retinal microvascular density and FAZ dimensions at the SCP and DCP. There was a weak relationship between ELM and EZ damage and visual acuity. 

The current study also showed that retinal microvascular density and FAZ dimensions in both the SCP and DCP were significantly lower in patients with diabetic retinopathy compared to those without diabetic retinopathy. A significant correlation was also observed between the vascular density in all ETDRS sectors and visual acuity. 

The most striking finding of the current study was the observation of the correlation between outer retinal disruption and retinal microvascular density at the level of both the SCP and DCP in diabetic retinopathy patients.

Evaluation of outer retinal layers or the ELM, EZ, and IZ bands on SD-OCT scans reveals important information about photoreceptor health [1]. Photoreceptors have the highest demand for nutrients and oxygen in the body [5]. Dual vascular supply by choroid and retinal circulation normally meets this high demand [9]. When requirements are not met, photoreceptors are at risk of degeneration and excitotoxic cell death [8].

Most of the metabolic and oxygen requirements of the photoreceptors are provided by choroidal diffusion [6]. The contribution of retinal circulation to photoreceptor blood supply through the DCP is about 15% [9]. The lack of autoregulation capability of the choroidal circulation results in an increased role of the DCP in photoreceptor nutrition under hypoxia [10]. On the basis of these data, it has been suggested that impaired retinal microvascular circulation in chronic hypoxic conditions such as diabetes may damage the outer retinal layers [11,12]. 

Studies investigating the association between macular perfusion and the integrity of the outer retinal layers in diabetic patients in the literature report conflicting results [11,12,16–18]. No significant relationship was found between FAZ and the dimensions and outer retinal layer status in patients with and without DME in some studies in which macular perfusion was evaluated by FFA [16,17]. On the other hand, other studies reported FAZ area enlargement and macular capillary nonperfusion areas in the DCP on FFA in cases with outer retinal disruption [11,18]. Enlargement of FAZ measurements at both the SCP and DCP levels and irregularity at the boundaries were reported in another study in which retinal microvascular perfusion parameters were evaluated by OCTA in patients with diabetic retinopathy. In addition, capillary nonperfusion areas were observed at both plexus levels in OCTA sections in the retinal areas corresponding to outer retinal disruptions on SD-OCT sections [12].

Outer retinal layers are hallmarks used to determine the health and integrity of photoreceptors. The findings of the present study show that there is a relationship between the degree of diabetic macular ischemia (DMI) and photoreceptor destruction. Similarly, there is a relationship between DMI grade and visual acuity. This supports the idea that visual impairment in DMI cases not accompanied by DME may be due to structural damage in photoreceptors.

The effect of a retinal microvascular perfusion defect on the outer retinal layer status has also been reported in retinal pathologies other than diabetic retinopathy. VD measurements in all sectors of the DCP have been reported to be significantly lower in Behçet disease patients with EZ and IZ damage than those without damage [19]. A significant relationship was also found between EZ damage and SCP and DCP VD measurements in patients with retinitis pigmentosa, but a similar relationship was not detected between FAZ measurements and choriocapillaris blood flow [20].

There was a relationship between the destruction of the EZ and IZ layers and VD measurements in both the SCP and DCP for all sectors in the current study. In contrast to the previous two studies, both the SCP and DCP FAZ measurements correlated with EZ and IZ destruction.

When the results of the studies in the literature and the present study are evaluated together, a significant relationship between the retinal microvascular density and FAZ dimensions in both the SCP and DCP and the development of outer retinal disruption can be seen. This supports the hypothesis that the role of the retinal microcirculation in the vascular supply of the outer retinal layers in hypoxic conditions is increased. Thus, the morphology and the integrity of the photoreceptors become more susceptible to retinal ischemia as a result. Macular ischemia via DCP perfusion defects can consequently lead to photoreceptor damage.

A limitation of this study is that healthy individuals who were matched by age and sex were not included in the study. The strong points of the study include the use of the most accurate and precise method to evaluate the retinal microvascular perfusion.

Studies that evaluate the association of retinal perfusion parameters with photoreceptor morphology at different stages of diabetic retinopathy will help to further clarify the pathogenesis of outer retinal layer destruction and determine the relevant factors.
